# Incidence of delirium and inadequacy of the clinical diagnosis in patients in intensive care

**DOI:** 10.1186/cc10947

**Published:** 2012-03-20

**Authors:** A Okada, R Azevedo, F Freitas, A Bafi, M Jackiu, M Assunção, B Mazza, F Machado

**Affiliations:** 1 Universidade Federal de São Paulo, Brazil

## Introduction

This study aims to assess the incidence, risk factors and impact of delirium on outcome and to analyze the concordance between the Confusion Assessment Method for the Intensive Care (CAM-ICU) and clinical diagnosis.

## Methods

A prospective observational study in a university hospital including patients over 18 years old, in the first 48 hours of ICU admission, with an expected ICU stay of at least 72 hours and signed informed consent. Pregnancy, cognitive impairment prior to admission, hepatic encephalopathy, Glasgow Coma Scale ≤9, active psychiatric illness, need for sedation or neuromuscular blockade, aphasia, foreign language, deafness and brain death were exclusion criteria. CAM-ICU was applied and doctors and nurses asked about the presence of delirium. Demographic data, SOFA score, mechanical ventilation and drugs used were determined. Patients were followed for 14 days or until discharge from the ICU. The agreement between CAM-ICU and clinical diagnosis was assessed using Cohen's kappa statistic (κ). Risk factors were assessed by a multivariate regression model.

## Results

In the 119 patients included, the incidence of delirium was 24.4% (29 patients) and time to development of delirium was 68.3 ± 63.6 hours. The agreement between clinical diagnoses and CAM-ICU was better for medical residents (Table 1). Patients with delirium had a longer ICU (10.83 ± 15.08 and 4.98 ± 9.57, *P *= 0.015) and hospital (36.93 ± 31.33 and 19.10 ± 19.48, *P *= 0.0004) length of stay, higher ICU mortality (13.79% and 2.22%, OR = 7.04 (1.22 to 40.7)) and hospital mortality (27.6% and 6.66%, OR = 5.33 (1.67 to 17.04)) than patients without delirium. Risk factors were: mechanical ventilation (*P *= 0.018, OR = 3.09 (1.21 to 7.86)) and APACHE II score greater than 8.5 (*P *= 0.011, OR = 5.35 (1.48 to 19.43)).

**Table 1:** κ values

Health provider	Delirium	Hypoactive
Attending physicians	0.530	0.019
Medical residents	0.615	0.018
Nurses	0.588	0.025

## Conclusion

Delirium had a higher incidence in intensive care patients and was related to longer hospital stay and higher mortality. Specific tests should be used for diagnosis, since the clinical suspicion has low sensitivity, especially in cases of hypoactive delirium and among attending physicians.
